# The expression of self-conscious emotions and their motivational and communicative function in early development

**DOI:** 10.1098/rstb.2025.0134

**Published:** 2026-09-24

**Authors:** Milica Nikolić, Robert Hepach

**Affiliations:** ^1^ Research Institute of Child Development and Education, University of Amsterdam, Nieuwe Achtergracht 127, 1018 WS Amsterdam, The Netherlands; ^2^ Department of Experimental Psychology, University of Oxford, Oxford, OX1 3EL, UK

**Keywords:** human development, self-conscious emotion, embarrassment, guilt, shame, pride, infancy, toddlerhood, early childhood

## Abstract

Self-conscious emotions, such as embarrassment, guilt, shame and pride, play a central role in managing reputation and interpersonal relationships. They are thought to emerge in the first years of life, with children's developing cognitive capacities and their growing appreciation of social norms and rules. Recent years have seen a rapid increase in research on self-conscious emotions in early life, supported by advanced methods including manual and automated micro-coding of non-verbal behaviour, depth sensor imaging and physiological measures. In this narrative review, we summarize this emerging evidence from developmental science, which suggests that self-conscious emotions may be observed earlier than previously thought—within the first months and years of life—and that they seem to serve both motivational and communicative functions from early on. Specifically, they motivate socially appropriate behaviours, such as helping others, while communicating adherence to social norms and rules. Further, we highlight current limitations in the field and recommend more experimental and longitudinal studies especially in infancy and toddlerhood, the integration of multi-modal data and the use of naturalistic tasks to advance our understanding of self-conscious emotions in early development.

This article is part of the theme issue ‘Mechanisms, development, phylogeny and functions of emotional expressions’.

## 1. Introduction

Research on the development of emotions in early childhood has flourished in recent decades, reflecting a growing recognition that emotions, alongside cognitive processes, are key drivers of human development [1]. Still, developmental research on emotions in the context of child development lags behind research on cognitive processes such as language and executive functioning [2]. When emotions are studied in young children, research focuses on a limited set of discrete emotions, e.g. anger, fear or joy, and their regulation [3]. By contrast, there is less research into so-called self-conscious emotions. Furthermore, and mirroring the broader field of affective science, research in early development has typically focused on children's perception and processing of others’ emotions using a child-as-spectator approach [4]. Here, children either passively view, or are asked to evaluate, others’ emotional expressions to make inferences and to draw conclusions about others’ minds. Informative as these studies may be to describe how emotions may be perceived and understood (e.g. ‘What does anger look like?’), this approach largely neglects the fact that children are not merely passive observers of their social world. Children are also active participants who interact with others and respond to personally relevant events around them [5]. These personally relevant events give rise to emotional states that inform and motivate both children's own behaviours as well as the behaviours of those around them. This interactive approach positions emotions as relational and situation-anchored (e.g. ‘How is the environment involved in children's anger and how does the expression of anger influence both the child and the environment?’) [6]. Investigating emotions from this perspective in early development allows us to shed light on (i) what young children might be feeling, (ii) what motivates their behaviours, and (iii) how they communicate their inner emotional states to others.

Self-conscious emotions play an important role in managing one's reputation and interpersonal relationships [7,8]. These emotions are thought to arise when one reflects upon the self from the perspective of others [8,9]. For example, embarrassment occurs when we feel socially exposed and realize that others may judge us (negatively) [10]. Guilt is experienced after one's actions have harmed another person and one recognizes that the other has been hurt as a consequence [11,12]. Displaying self-conscious emotions in contexts of mishaps or transgressions is theorized to help the expresser to show awareness of having broken social norms and rules, as well as in demonstrating concern for those norms and for others’ evaluations. In this way, such displays may help individuals manage their social standing and maintain important social relationships [10]. Studying these emotions in early childhood thus provides a unique opportunity not only to infer how children might feel in contexts of social violation and transgression, but also to probe their emerging understanding of their own and others’ minds as well as social rules and norms.

To date, self-conscious emotions have primarily been studied in older school-aged children and adolescents. Research has largely relied on questionnaire data based on self- or parent-reports to investigate self-conscious emotions (e.g. [13,14]). However, subjective experiences captured by self- or other-reports constitute only one of several components of emotions. They are also limited in that they cannot be used with pre-verbal or just-verbal children. Therefore, relying solely on self- or other-reports can constrain our understanding of emotions in early childhood and hamper empirical research and the development of comprehensive theories of emotional development [15]. In young children, studying other aspects of emotion, such as non-verbal expressions and physiological responses, may be more informative for understanding their emotional development.

Emotional expressions, including facial displays and body posture, as well as visible physiological reactions such as blushing, are increasingly used as valid and reliable measures of self-conscious emotions [16–18]. Although non-verbal expressions of self-conscious emotions are complex and involve not only facial expressions, but also gaze and head direction as well as body posture, it has become possible to study these emotions in a more fine-grained and reliable manner with the recent developments of detailed coding systems [18] as well as novel automated measurement tools [19,20]. Although these behavioural and physiological measures are not without limitations (e.g. subjectivity of manual coding, limited consideration of context-dependence in automated coding, artefacts and limited specificity in physiological measures), they hold considerable promise for advancing our understanding of self-conscious emotions even in the earliest ages of life.

In this narrative review, we address the development of self-conscious emotions in infancy (from birth to 2 years of age) and early childhood (to 8 years of age)—the developmental period in which self-conscious emotions emerge and undergo rapid change as new cognitive capacities develop and children encounter increasingly complex social situations [9]. The most recent developmental research sheds light on (i) when these emotions emerge, (ii) how they are expressed, and (iii) what effect they have on children's subsequent behaviours. We focus on infancy and early childhood, a previously understudied period in the study of self-conscious emotional development, largely owing to the methodological limitations mentioned above.

**Figure 1.**
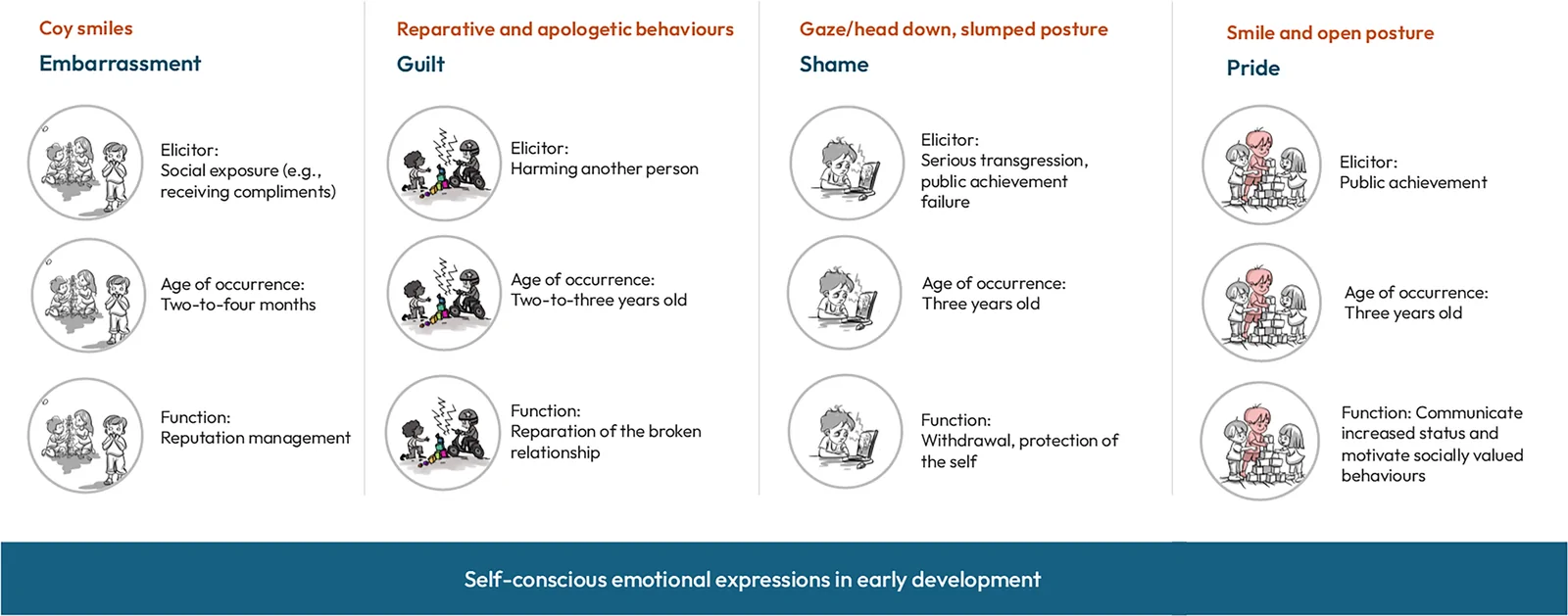
An overview of the state-of-the-art in self-conscious emotional development research. *Note:* the illustrations are the work of Lysa Adams.

We review developmental research on four specific self-conscious emotions— embarrassment, guilt, shame and pride—which have received increased attention over the past decades. We group existing research into two broad categories: (i) the development of emotional expression—when and under which circumstances self-conscious emotions are expressed in the face and body in early child development, as well as (ii) the motivational and communicative functions of self-conscious emotional expressions—what subsequent behaviours these emotions motivate in the expresser and what they communicate to others. A summary overview of our approach is presented in figure 1.

## 2. The development of self-conscious emotion expression in early childhood

Prototypical expressions of so-called ‘basic’ or ‘typical’ emotions primarily involve facial muscle movements, but in contrast, self-conscious emotional expressions may include gaze and head direction as well as body posture and movements, in addition to facial muscle movements [17,21]. Therefore, focusing solely on facial expressions may not be informative enough [22] when capturing self-conscious emotions. Body movements and posture, gaze/head direction, as well as their combination with facial expressions, have increasingly been used to index self-conscious emotions [20,23,24]. For example, positive self-conscious emotions have been found to expand upper body posture in adults and to some extent in 5- to 10-year-old children, whereas negative self-conscious emotions do not [25,26]. Consequently, recent research has employed precise manual or automatic frame-by-frame micro-coding to capture various aspects (e.g. a smile, gaze aversion, body touching and changes in body posture) of (self-conscious) emotional expressions in videos. This recent approach has enhanced the accuracy of capturing emotional expressions and has built on past research, which predominantly relied on subjective coding where coders watched videos and subjectively judged the presence or absence of self-conscious emotions or used carers’ reports.

### (a) Embarrassment

The first self-conscious emotion thought to emerge in infancy is embarrassment [27], which has also been referred to as self-conscious shyness, positive shyness or coyness [28–30]. Infants express embarrassment in response to social exposure, such as receiving compliments in face-to-face interactions [29,30] or being exposed to a mirror [27]. A prototypical expression of embarrassment in infancy, similar to the embarrassed smile in adults, is a coy smile, which consists of a smile during which a child looks away and/or averts their head, sometimes followed by a face touch [23,27,30,31]. Unlike in adults, where the embarrassed smile is often controlled (with lips pressed together) [21], the coy smile in infants and young children typically consists of an open smile or a Duchenne smile during which gaze and/or head aversion occurs [23,29,30].

Early studies of embarrassment displays in infancy found that infants from Western countries, in the second half of the second year of life (typically between 18 and 24 months), start displaying embarrassment when exposed to their own picture in the mirror or when exposed to the attention of others [27,31]. The emergence of embarrassment in the second year of life has been linked to the emergence of self-awareness, commonly assessed with a mirror self-recognition task in which infants who touch a mark placed on the face are taken to have acquired a concept of the self as an object of others’ attention [27].

More recent observational studies, using continuous micro-coding of coy smiles with high inter-rater reliability (e.g. [30]), have shown that infants from Western countries display coy smiles already in the first months of life when exposed to social attention, for example, when receiving compliments during social interactions [23,29,30,32]. These studies raise the possibility that exposure embarrassment, or at least embarrassment-like responses to social exposure, may be present earlier than previously thought. At the same time, experimental studies with adequate control conditions are needed to determine whether coy smiles produced in these situations are indeed specifically elicited by social exposure, as opposed to other aspects of compliment situations, such as cognitive overstimulation or the generally positive tone of social or non-social situations.

Somewhat later in development, typically around 3 to 4 years of age, children begin to consider how they may be perceived by others, which has been linked to the emergence of evaluative embarrassment [33]. This type of embarrassment is elicited when one realizes that others can evaluate or judge one negatively. In addition to facial expressions, evaluative embarrassment is thought to be accompanied by physiological blushing, that is, reddening of the cheeks (and sometimes the forehead, ears and neck), which can be indexed by increased blood flow or elevated skin temperature in these regions [34].

Blushing in children has only recently become a focus of empirical investigation. Initial results show that children as young as 3 years exhibit blushing in embarrassing situations, in a manner comparable to adults. For example, increases in blushing has been observed during public performance or when watching one's own performance in front of an audience, compared with a neutral baseline [35,36], or to situations without an audience [37] in children aged 3 to 5 years from Western countries. However, little is known about whether evaluative embarrassment can occur earlier than the age of three, or whether blushing may occur with exposure embarrassment in infants or toddlers.

One recent fMRI study on blushing suggests that blushing does not seem to require the engagement of complex socio-cognitive skills, but may instead constitute a relatively automatic response to social exposure. This raises the possibility that younger children, or even infants, could show blushing in response to social exposure [38]. A preliminary study with 10 two-month-old infants from a Western country measured facial temperature during interactions with the mother and with a stranger, and found greater overall increases in facial temperature in interactions with the stranger than with the mother, suggesting some sensitivity to social context [39]. However, this temperature increase occurred across a range of social situations, including play and neutral interactions, and was not specific to social exposure. Thus, the possibility that young infants blush specifically in response to social exposure remains to be tested in future experimental studies.

Findings regarding gender differences in embarrassment are mixed. Some studies report no differences in coy smiles between boys and girls in the first months of life [29], whereas other studies find that girls display more coy smiles than boys in the second year of life [27]. This pattern may point to a role of experience and culture in shaping emotional responding in ways that are consistent with gender roles, although such differences have not been observed for blushing in early childhood [35,36]. More research in early development is needed to determine whether gender differences in embarrassment are present from early in life and how they change with children's age.

### (b) Guilt

The self-conscious emotion guilt is a negatively valenced emotion that typically follows accidental or intentional harm inflicted on others, and involves concern for the harmed person and a wish to repair the wrongdoing [11,40,41]. Guilt encompasses the feeling that one has done something wrong, with the focus on the wrongful action rather than on the whole self as bad [41]. There does not appear to be a clear prototypical non-verbal expression of guilt, especially at the level of facial displays [21,42], although guilt has been related to eyebrow lowering and neck touching [43]. Because guilt involves a motivation to repair, past studies with children have typically studied the expression of guilt through reparative behaviours [40].

The first studies on the expressions of guilt in 2- to 4-year-old children from Western countries were conducted in the 90s using a ‘broken toy’ task in which children are led to believe they have damaged the experimenter's valuable possession (i.e. a teddy bear that has been rigged so that its arm falls off when the child tries to play with it) [40,44]. Consistent with the phenomenology of guilt, some children displayed reparative behaviours and accepted responsibility for the mishap. Similar patterns have also been observed more recently in 18-month-old infants from Western countries [45,46]. This pattern suggests that guilt-like behaviours may emerge relatively early in child development; however, experimental studies are needed to distinguish guilt from other processes that may arise when someone is harmed, like sympathy for the victim or more general prosocial tendencies. While helping a victim whom one has previously personally harmed might be indicative of guilt, helping a victim regardless of who caused the harm is considered to reflect sympathy. More recent experimental work has begun to dissociate guilt from sympathy in young children from Western countries. In one study, 2- and 3-year-old children played a game in which they were made to either believe that their own actions had accidentally broken another person's block tower (guilt condition) or saw another adult accidentally break the same tower (sympathy condition) [47]. Three-year-old, but not 2-year-old children displayed more reparative behaviours in the guilt condition compared with the sympathy condition, suggesting that children's motivation to repair was especially pronounced when they themselves were responsible for the harm. Crucially, the study included two control conditions in which either the child's (or the adult's) actions did not cause harm to others, and reparative behaviours were lower in these conditions than in the guilt condition, as expected. These results are consistent with the view that guilt emerges during the third year of life, when children help and repair more after they have caused harm to others. Before this age, guilt-like reparation after another person is harmed may also occur, but may possibly reflect a more general motivation to help based on sympathy.

Most past studies have not examined or reported on robust gender differences in guilt-related behaviours in the second and third year of life [45,46], with the exception of one study that found more guilt-related behaviours in 2-year-old boys compared with girls [40]. Current evidence thus does not yet support a consistent pattern of gender differences in guilt-related behaviours in early childhood.

### (c) Shame

Shame is a self-focused, negatively valenced self-conscious emotion that appears after committing a transgression and is thought to require a stable self-representation, as it involves the feeling of the whole self being a failure [41]. It is typically expressed through downward head and gaze direction and lowered upper-body posture (i.e. slumped shoulders and a narrowed chest) [21,40,48]. With regards to its development, shame is different from guilt in the following ways: (i) the experience of shame is not necessarily a direct consequence of having harmed others, but can result from violating norms and standards more broadly; therefore, it may occur with or without transgressions involving others [49]; (ii) the feeling of shame is exacerbated in the presence of an audience (uninvolved observers), which is not so much the case when experiencing guilt [50]; (iii) children understand harm as a consequence of their own actions at younger ages than they understand the violation of social norms as such; (iv) while guilt focuses on a specific wrongful behaviour that has caused someone harm, shame focuses on the global self [41]; as a result, shame is thought to require a stable self-representation, whereas this may be less critical for guilt. Taken together, these considerations suggest that shame develops later than guilt in early childhood.

The first studies on the expressions of shame (and guilt) in 2- to 4-year-old children from Western countries used the broken toy task and found that, consistent with the phenomenology of shame, some children displayed avoidant behaviours, such as gaze and head aversions and avoidance of the experimenter [40,44]. Similar patterns have been observed more recently in 18-month-old infants from Western countries [45,46] and in a longitudinal study including children from 22 to 45 months of age [51].

These studies also indicate that toddler girls tend to show more shame-related expressions than boys [40], and that even when gender differences are not evident in early toddlerhood, girls begin to show more avoidant behaviours than boys in late toddlerhood and the preschool years [51]. However, one study in toddlers did not find gender differences in shame-related expressions [45], suggesting that evidence is not consistent.

Extending this work beyond moral contexts involving harm to others, studies have also examined shame in non-moral settings, such as public performance failure [52,53]. Similar to moral settings (i.e. broken toy mishap), 2-to 5-year-old children [52], and 3- to 5-year-old children from Western countries [53] displayed shame through avoidance behaviours after a failure on a performance task. Interestingly, children expressed more shame in the performance-failure situation than in the broken toy mishap [53], suggesting that failure on performance tasks may be a particularly salient situation in evoking shame in young children. This aligns with earlier work showing non-verbal expressions of shame, including gaze and head aversion as well as more slumped upper-body posture, in 3-year-old boys from Western countries after failing an easy task, such as solving an easy puzzle [54] or after failing an unsolvable task presented as ‘easy’ compared with a ‘difficult’ task [55]. In these studies, girls also showed more avoidance behaviours than boys [51,54], suggesting that across both moral and performance-failure situations, girls tend to display more shame than boys.

While much of the earlier work on shame relied on subjective or objective manual coding of behaviour to capture the expressions of shame, more recent work has begun to use depth sensor imaging to automatically measure shame-like emotions through body posture changes, in particular the slumping of the upper-body posture [20,56]. Depth sensor imaging technology is based on a specialized camera that uses infrared light to track participants’ body points and assess postural changes (for technical details, see [20,25]). For example, one study found some evidence for lowered upper-body posture in 4- to 5-year-old children from Western countries both after they failed to help someone in need and after they failed to complete their own goal [57]. Similarly, 3- to 5-year-old children from Western countries exhibited lowered upper-body posture after making unjustified requests from an adult (i.e. asking for a sticker that they did not need but that the adult needed) compared with justified requests [58]. Additionally, older children from Western countries aged 7 to 9 years showed lowered upper-body posture after unintentionally disadvantaging a peer in a resource allocation task [59].

Taken together, these studies provide initial experimental evidence that shame-like emotions can be observed as early as the age of 3 years. However, as with guilt, naturalistic observations suggest that shame-like behaviour may already occur in the second year of life. Such observations, however, cannot definitely determine whether these behaviours are the result of shame or other related emotions. For example, withdrawal and avoidance may also arise from the experiences of fear [60]. Future studies with younger children should employ experimental designs with appropriate control conditions to exclude this possibility. For example, comparing young children's behaviours across social situations that might evoke social fear (e.g. interaction with a stranger), while manipulating elicitors of shame (e.g. moral transgression or performance failure) could help determine whether avoidance and lowered upper-body posture are driven primarily by fear or shame.

### (d) Pride

Pride is a positive self-conscious emotion that, similarly to other more negatively valenced self-conscious emotions, is thought to appear in the third year of life when children acquire stable self-representations and can compare their own behaviour with external standards [61]. We experience pride when we attribute success in a personally relevant area to our own efforts [8]. Extant past research has documented that pride has a prototypical non-verbal expression that includes a smile in combination with an expanded body posture, head tilted slightly back and arms akimbo [62]. Interestingly, this research on the prototypical expression of pride was based on young children's behavioural reactions to success [8].

The first studies of pride in young children investigated 3-year-old Western children's reactions to success in a difficult task in comparison with an easy task [54,55]. Pride was indexed through a smile and expanded body posture, along with positive self-evaluations (e.g. ‘I did it!’). These studies found that pride expressions were more likely to occur in 3-year-old children after solving a difficult task compared with an easy task. One recent study replicated these findings, showing that preschool-aged children express more pride after the completion of a difficult task compared with an easy task [63]. In addition, this study also found expressions of pride to be elicited in a helping task in which young children who spontaneously helped an adult experimenter to retrieve a dropped crayon showed more pride than children who were prompted to do so. Similarly, 3- to 6-year-old Western children expressed pride after successfully solving a task in the presence of another person [64]. Another recent study found that young children aged 3 to 5 years appear to experience a mix of self-conscious emotional arousal, including both pride and embarrassment, when viewing a video of themselves successfully solving a puzzle [37]. This set of studies suggests that pride can be evoked in a range of social achievement situations starting at the age of 3 years.

Recent studies have also used depth sensor imaging to automatically measure pride-like emotions through changes in body posture, particularly expanded upper body posture [20,56]. For example, in a recent study with 5-year-old children from Western countries, expanded body posture was observed when children helped the experimenter at a personal cost (they had to restart their activity). This was not the case when helping was not costly (children could leave their activity with no loss to progress) or when they merely watched the adult complete their own task [65]. This suggests that incurring a cost to help may evoke pride in young children. In line with this study, another recent study found that Western 5-year-olds, but not 2-year-olds, showed expanded body posture indicative of pride when they helped another person, but not when someone else helped. This distinction highlights that pride occurs when we attribute success to our own efforts, differentiating it from other emotions [66]. Interestingly, 2-year-olds exhibited expanded body posture regardless of who provided help, suggesting that they may experience a more general positive arousal, rather than pride specifically, when seeing someone receive help. Moreover, postural elevation effects in 5-year-old children were especially pronounced when the child participant was observed by 2 adults, indicating that the presence of an audience may further enhance pride.

Although these findings suggest that 2-year-old children might not experience pride after showing prosocial behaviours, another study showed that children exhibit pride reactions after a success as early as 20 months of age [67]. One possible explanation for these mixed findings is that pride may only just be emerging around the age of 2 years and may not yet be easily elicited across a broad range of social situations, as appears to be the case from 3 years onward. At this early age, clear achievement contexts (e.g. a success on a structured task with an explicit goal) may be more likely to elicit pride than more spontaneous prosocial helping, where the goal is less clear to very young children. More experimental studies that systematically vary the elicitors of pride (e.g. costly helping, performance success on difficult tasks or in the presence of others) at these early ages are needed to provide more robust evidence on the early development of pride and the conditions under which it is evoked. In addition, only two of the above studies on pride expressions have examined gender differences and they did not find any differences between girls and boys [54,65], suggesting that, unlike some other self-conscious emotions, pride may be experienced and expressed similarly in young girls and boys.

## 3. Motivational and communicative aspects of young children's self-conscious emotional expressions

Why do children display self-conscious emotions early in development? Theories and empirical studies in adults suggest that self-conscious emotions motivate and regulate one's own behaviours while also serving communicative functions that support managing their reputation and maintaining cooperative relationships [10,61]. For example, as well as communicating directly to nearby companions, expressions of embarrassment after a mishap or expressions of shame after a serious transgression may alert the expresser themselves that they have behaved inappropriately in front of others. This may thereby promote social harmony by demonstrating that an individual is aware of social norms and rules and that they care about others’ opinions of them [7,10]. Expressions of guilt may promote apologetic behaviours and forgiveness [11,12,68], while expressions of pride may signal to others an increase in social standing or expertise and competence [8]. Furthermore, self-conscious emotions may serve to reinforce socially appropriate behaviours [68]. For example, the rewarding experience of pride may motivate an individual to persevere, and the punishing experience of embarrassment or shame may deter individuals from engaging in wrongdoings. Do self-conscious emotional expressions serve similar purposes early in human development?

Given that young children have relatively limited experience and understanding of the rules and conventions of their social world compared with adults, it is important to avoid drawing direct conclusions from theories and empirical evidence based on adult studies. Instead, the focus should be on developmental theories and empirical studies with young children as participants. While theories of self-conscious emotion development in early childhood exist [33,69,70], they do not always directly address the question of what functions self-conscious emotions serve in early development. However, Stegge [70] argues that self-conscious emotions allow children to achieve key developmental milestones in social development. For example, shame helps children develop a stable self-concept to internalize social rules and norms. It also motivates children to conform by avoiding shameful experiences. Similarly, blushing—the hallmark expression of a self-conscious emotion—fulfils the adaptive function of regulating children's social interactions by showing commitment to social rules and norms, which may prevent others’ negative reactions and social exclusion after a transgression. Empirical research on whether and how children's self-conscious emotional expressions serve motivational, regulatory and communicative functions is still limited. In the following, we review this research with respect to embarrassment, guilt, shame and pride.

### (a) Embarrassment

Embarrassed or coy smiles, as well as blushing, are theorized to occur when an individual recognizes that they have committed or will commit a violation of a social convention [10,70]. This response communicates to others that the embarrassed individual cares about these norms and others’ opinions of them. It may therefore attenuate negative evaluations by others and evoke affiliative behaviours from the social environment [71]. Research in adults supports this regulatory and communicative role of embarrassment displays and blushing in situations involving mishaps and transgressions [72–75] (although context-dependence and potentially negative social consequences of blushing have also been discussed [76]).

Less is known about the motivational and communicative functions of embarrassment in early childhood. A recent study comparing younger (3- to 5-year-old) and older (8- to 12-year-old) children with adults from a Western country examined whether experiences and expressions of embarrassment, induced by watching their own singing performance, differed as a function of the presence of an audience [37]. The findings showed that all participants (including younger children) expressed more embarrassment through blushing (but not through coy smiles) in the presence of an audience compared with its absence. The increase in expressed embarrassment in the presence of others may suggest that even young children are sensitive to social–evaluative contexts and may care about how others perceive them. These findings are consistent with the idea that embarrassment already serves to manage social standing and to appease others in early childhood, although more work is needed to establish its effects at these early years.

### (b) Guilt

Guilt involves an evaluation that one's own behaviour, not the whole self, was wrong. Guilt, therefore, motivates repairing the wrongdoing or the relationship with those who were harmed. This, in turn, appeases others and elicits cooperation [11,12]. Research in adults confirms these effects of guilt on behaviours of transgressors and their environment [43,77]. Research on the effects of guilt on subsequent behaviours in childhood has primarily focused on prosocial behaviours, with the idea that guilt enhances prosocial behaviours [78]. One study with Western toddlers investigated whether their self-conscious emotional reactions to breaking the experimenter's ‘favourite’ toy related to their subsequent helping behaviours towards the experimenter whom they had previously accidentally harmed [45]. Toddlers who displayed guilt-like behaviours, including trying to repair the toy and confessing to the experimenter, demonstrated more frequent and faster subsequent helping behaviours in another unrelated task. Another recent study extended these findings to a broader age range of Western children, including 2- to 5-year-olds [24]. This study provided corroborating evidence that guilt-like behaviours are associated with more helping across toddlerhood and early childhood. Together, these studies suggest that starting at the age of 2 years, guilt is associated with more prosocial behaviours towards the victim (see also [47]).

One experimental study with older 6- to 10-year-olds children from Western countries induced guilt by leading children to believe that they had transgressed, causing distress to a peer, compared with not having caused distress to a peer in the control condition [79]. Children in the experimental condition, that was meant to induce guilt through the concern for the harmed distressed peer, were more likely to share their stickers and write a note to the distressed peer, suggesting that causing distress to others, specific to guilt, promoted prosocial behaviours [79]. Another experimental study with young Western children aged 2 and 3 years used pupil dilation as an index of emotional arousal to test whether young children in a guilt-evoking condition would be especially prone to helping [80]. In this study, after accidentally harming a victim, 3-year-olds (and to a lesser extent, 2-year-olds) were especially motivated to repair the harm themselves, and their emotional arousal reduced after doing so, but arousal remained high if someone else repaired the harm the child had caused. These differences were not present when someone else, rather than the child, caused the harm. This suggests that 3-year-old children (and possibly even 2-year-old children) can recognize when they have transgressed, probably leading to feelings of guilt, which then motivate reparative behaviour.

Extending beyond individual guilt, one recent study showed that children, starting at the age of 5 to 6 years, show group-based guilt in response to transgressions committed by in-group members and engage in prosocial behaviours such as apologizing and helping to repair the harm caused by their in-group [81], reflecting children's developing capacities to understand the complexities of the social world (see also [82]). This study, conducted with Chinese children, is one of the few to examine young children's self-conscious emotions in non-Western samples, and suggests that at least some motivational and communicative functions of self-conscious emotions may be shared across cultures in early childhood.

### (c) Shame

Shame is theorized to serve different functions from guilt because the actions it motivates are different from the actions motivated by guilt [40]. Given that shame involves a feeling that the whole self and reputation are threatened, individuals who experience shame engage in escape behaviour to protect the self from additional scrutiny. Therefore, children and adults who experience shame will hide from others and shrink in size (by averting their gaze, lowering their posture) to lessen the risk of social exclusion and shunning [48,83].

Similar to work on guilt, research on the effects of shame on children's subsequent behaviours has primarily focused on prosocial behaviours, with the idea that shame inhibits such behaviours [24,40,45]. Two studies investigating Western children's emotional responses to breaking the experimenter's ‘favourite’ toy captured shame-like behaviours [24,45]. Toddlers who showed shame-like responses, such as avoiding the experimenter and rarely confessing or attempting to repair the toy, subsequently showed less frequent helping in an unrelated situation. This effect was consistently found across children at toddler age and at preschool age. In sum, this work suggests that shame, which is expressed through avoidance and withdrawal, may subsequently reduce prosocial behaviours in young children.

However, these studies were observational and did not include control conditions that would rule out alternative explanations. For example, reduced helping might also arise from general negative affect or distress about the toy being broken, regardless of whether the child feels personally responsible. Experimental studies that compare situations in which the experimenter's ‘favourite’ toy breaks and the child is versus is not led to believe that they caused the damage would help to determine whether reduced prosocial behaviours are specifically owing to shame, rather than to general distress or other processes.

### (d) Pride

Pride expressions are theorized to inform an individual and their environment about their currently increased social standing and to motivate efforts towards socially valued behaviours, e.g. being competent or being helpful to others [61]. Empirical research in adults supports these ideas, showing that proud participants are more likely to attain high status and are motivated to achieve high standards in future tasks [84–86].

Several recent studies have addressed the motivational aspects of pride in young children. In the first study to relate pride expressions to prosocial behaviours in young Western children aged 3 to 5 years, children's pride expressions following an achievement were associated with their helping behaviours and making amends [63]. Another experimental study with Western children of the same age group built on these findings and showed that inducing pride (along with other positive moral emotions) by engaging children in a task involving helping an adult experimenter tidy up a table (compared with a control group that did not engage in a prosocial act) promoted helping, sharing and comforting [87].

Whether it is the non-verbal expressions themselves that inform the expresser of their achievement and motivate subsequent behaviour, or whether it is the associated subjective experience of pride (which may occur with or without clear non-verbal expressions) that drives children's subsequent behaviours is difficult to determine without directly manipulating expressions and experiences. Such manipulations are particularly challenging in very young children because of their limited verbal and introspective capacities. Nevertheless, in line with functional accounts of pride, these findings suggest that children's pride (whether expressed, experienced or both) can motivate socially valued behaviours, such as helping and sharing with others, from as early as the age of 3 years. Whether similar effects occur earlier in development has, however, not been studied to date.

More recent experimental studies have also investigated whether pride expressions serve to communicate one's increased social standing by systematically varying the presence of an audience. One study did not find increases in pride expressions (indexed through facial and postural measures) when 3- to 5-year-old children from a Western country watched a video of their own success on a performance task in the presence of an audience (versus in its absence) [37]. By contrast, another study using body posture to index pride found that 5-year-old Western children showed more expanded body posture (similar to pride) after helping another person when an audience was watching [64].

These mixed findings may have several explanations. One possibility offers a developmental account: children at around 5 years, but not earlier, may increasingly experience and express pride in the presence of others to manage their own reputation (i.e. implicit reputation management) and the desire for others’ recognition of their specific achievements (i.e. explicit reputation management) becomes more salient as children grow older [88,89]. Another possibility lies in methodological differences, such as indexing pride through facial plus postural changes versus postural changes alone or using different types of achievement tasks across studies. Further research that systematically varies age, type of pride elicitor and measurement modality is needed to clarify the conditions under which pride expressions begin communicating social standing in early childhood.

## 4. General discussion

The study of children's expressions of self-conscious emotions started at the end of the twentieth century with research on infants’ embarrassment [27] and young children's guilt [40,44], shame [40,44,54,55] and pride expressions [54,55]. Theoretical accounts at the time posited that the majority of self-conscious emotions appear at the end of the third year of life (except exposure embarrassment, which should occur at the end of the second year of life).

However, we now know, based on more recent empirical research using advanced techniques to index self-conscious emotional expressions (e.g. manual and automatic micro-coding of behaviours, depth sensor imagining and physiological measures), that children's self-conscious emotional expressions can be captured earlier in development than previously thought. For example, coy smiles of embarrassment have been observed in the first months of life [23,30], and guilt and shame expressions have been documented in toddlers as young as 2 years of age [12,46,52]. These expressions closely reflect those of older children and adults and are elicited in situations theorized to evoke these emotions (e.g. embarrassment during social exposure, guilt and shame after a transgression and pride after success on a difficult task). Therefore, there is now growing agreement among developmental researchers that children are capable of experiencing and expressing at least rudimentary forms of self-conscious emotions in the first years of life.

However, to provide more robust evidence, experimental studies with relevant control conditions to exclude factors other than the experience of self-conscious of emotions, such as general positive or negative tone of social situations, are needed to ensure that these self-conscious emotions indeed underlie children's observable behaviours. It is worth noting that while the emotions of young children, especially infants and toddlers, might not closely mirror those seen in adults, they do seem to affect young children's own behaviours. This highlights the importance of studying these emotions in early development at an age before these emotional experiences may become more adult-like as children become more immersed in their social environment.

What do these findings imply for developmental theories of self-conscious emotions? First, self-conscious emotions are thought to be cognitively complex and require sophisticated socio-cognitive skills, including self-awareness, perspective taking and the understanding and internalization of social rules and norms [33]. On this view, such emotions are expected to emerge relatively late in development.

However, more recent theoretical accounts in developmental psychology suggest that these emotions can emerge at an earlier age, presumably because the experience of these emotions does not require that the relevant complex socio-cognitive skills be explicit. Rather, implicit (socio-)cognitive abilities—automatic processes occurring outside of conscious awareness [90]—may be sufficient for these emotions to occur [91–93]. Recent empirical evidence suggests that socio-cognitive skills, such as self-awareness [93], mentalizing [94], perspective taking [95] and understanding of moral transgressions [96], are acquired implicitly at a very early age. This may help explain why we may observe self-conscious emotions already in infancy and early childhood.

Consider embarrassment as an example. It has often been thought that experiencing and expressing embarrassment require explicit self-awareness—a concept of the self as a social object [27]. However, recent observations of embarrassment-like displays in infants who do not yet show explicit self-recognition suggest that forms of embarrassment may be present in the first months of life. At these early ages, infants may possess an implicit sense of self, which could be sufficient to support rudimentary forms of self-conscious emotions [91,93].

In addition, some theories of emotion argue that experiencing a specific emotion requires having a concept of that emotion [97]. While these concepts need not be explicit, language is often considered to play a major role in the development of emotions [98]. On this view, children are thought to be able to experience certain emotions only once they have learned the corresponding emotion word and understand when and why the emotion typically occurs, which typically develops around the school-age period. However, other theories suggest that children may be able to display certain emotions before acquiring explicit concepts for them [99]. In line with this, children explicitly understand self-conscious emotions around 5 or 6 years of age [100,101], but may develop a relatively mature, though not yet articulated, understanding of these emotions at a younger age [102]. At the same time, reviewed evidence shows that children display behaviours consistent with self-conscious emotions much earlier, suggesting that a fully mature, explicit understanding of an emotion may not be required for children to experience that emotion.

What can this recent research and theorizing on early self-conscious emotions tell us about children's social development? First, it may provide insights into children's emerging social understanding, including the development of self-presentational concerns and sensitivity to moral and social standards. For example, a study measuring body posture as an indication of young children's positive pride-like emotions found a developmental shift in prosocial motivations from 2 to 5 years of age [66]. For 2-year-olds, helping another person and watching another person provide help resulted in equally positive emotions. However, 5-year-olds showed greater postural elevation when they themselves helped another person compared with when someone else was helping. In addition, these children exhibited greater postural elevation, indicating stronger positive emotions after helping others in the presence of an audience. This pattern suggests that younger children have an intrinsic concern that others who need help receive it, regardless of who provides it, whereas older children seem to have an additional, strategic self-presentational motivation to help, linked to how they are perceived by others.

Other studies manipulating the presence of an audience show similar patterns: self-conscious emotional responses are stronger in young children when an audience is present, pointing to the emergence of self-presentational concerns in early development, between 3 and 5 years of age [37,64]. For instance, two studies found that embarrassment [37] and pride [64] displays were heightened when embarrassing or achievement situations were witnessed by others. This suggests that young children are sensitive to the evaluative stance of others and care about how they are seen, which can amplify the experience and expression of self-conscious emotions [37].

At the same time, these expressions can have positive social effects on observers, helping the expresser navigate their social standing [7,10,61]. The fact that even young children show audience-sensitive displays of self-conscious emotions raises the possibility that they already use self-conscious emotions, at least to some extent, to manage their reputation in context-appropriate ways (e.g. appeasing others with embarrassment displays after they have done something embarrassing or raising in their social standing in the eyes of others after they have achieved a socially desirable goal).

Second, this research helps to clarify the motivations behind young children's social behaviours. Studies on shame, guilt and pride have shown that evoking these emotions may lead to varying levels of prosocial behaviours, including helping, sharing and comforting, in young children [79,87]. These findings are consistent with the idea that self-conscious emotions already contribute to regulating social behaviours in early childhood, in ways that partly parallel their functions in adults. At the same time, this emerging work points to potential applications: a better understanding of how self-conscious emotions motivate prosocial actions may inform the design of early preventive programs aimed at promoting healthy social development.

## 5. Limitations and future directions

This narrative review suggests that significant advances have been made in the study of self-conscious emotions in early development but our understanding remains incomplete. However, our knowledge is often based on a limited number of mostly correlational studies, which precludes robust conclusions. Furthermore, existing studies often rely on different measures and use different situations to elicit particular emotions, and they test children of varying ages, which can lead to inconsistent results. For example, an increase in pride-related expressions indexed by expanded upper-body posture in the presence of an audience has been observed in 5-year-olds in a helping context, but not in younger children's non-verbal expressions of pride including a smile, direct gaze and head tilt or expanded chest when watching a video of their own successful performance. To resolve such mixed findings, future studies should systematically employ multiple emotion-eliciting situations across different ages and compare different aspects of non-verbal expressions within the same studies. In addition, much of the reviewed research is correlational and cross-sectional. Therefore, more experimental and longitudinal studies are needed to better understand the causes and developmental trajectories of these emotions.

Considering the important role of socio-cognitive skills as prerequisites for these emotions, more research should investigate how and whether the development of socio-cognitive skills coincides with the emergence of self-conscious emotions. For example, do changes in the developing sense of self change how and when embarrassment is observed? Or does an increasing understanding of moral transgressions affect the expression and occurrence of guilt or shame? Considering that current research suggests that implicit socio-cognitive skills may be sufficient for self-conscious emotions to occur, and given that non-human primates possess (precursors of) many of these capacities [103,104], together with evidence that self-conscious emotions serve to repair social relationships and that many non-human primates show abilities to maintain social relationships and group cohesion through conflict intervention, reconciliation and resolution [105], it is now possible to ask whether non-human animals might be capable of rudimentary forms of self-conscious emotions [106].

Also, considering the importance of self-conscious emotions for social functioning, future studies should examine heritable and environmental factors influencing their development. For example, it is important to understand how carers socialize self-conscious emotions, depending on heritable factors such as temperament. Finally, very few past studies have used multi-modal data, including behavioural observations, physiological measures and carers’ or self-reports. Relying on only one modality does not capture multiple components of emotions. Therefore, more studies using multi-modal data are needed to gain a comprehensive understanding of self-conscious emotions in early childhood.

Some limitations that are widely recognized in affective science also apply to the study of emotions in young children. For example, capturing non-verbal expressions can be challenging because manual coding by human observers is inherently subjective. Such coding is also labour-intensive and may require specialized and sometimes costly software. Automatic coding of self-conscious emotions can reduce subjectivity, but many emotion researchers agree that current software is not yet sufficiently sensitive to reliably distinguish complex and context-dependent expressions.

Physiological measures bring their own challenges. Movement artefacts can reduce the reliability of these measures, and although this can be mitigated to some extent (e.g. by limiting movement or using advanced filtering), questions about specificity of physiological measures remain. Taking blushing as an example, increases in cheek blood flow and temperature are often used as indices, but it is not always clear whether such changes reflect blushing *per se* or are driven by other unrelated factors, such as movement and external temperature conditions.

A further important limitation, especially salient in research with young children who cannot reliably report their emotional experiences, is the uncertain mapping between non-verbal expressions and specific emotional states. When investigating the motivational aspects of children's emotions by relating children's expressions to their subsequent behaviour, it often remains unclear whether it is the non-verbal expressions themselves, the associated subjective experience, or the awareness of these expressions that primarily drives behaviour.

More general limitations in developmental science also apply to the study of self-conscious emotions. Most existing research has been conducted with children from Western contexts, which limits the generalizability of findings. We know that culture plays an important role in how and when self-conscious emotions are experienced and expressed [26], and more research in non-Western cultures is urgently needed. For example, some self-conscious emotions, such as shame, are more socially accepted in East Asian cultures [107]. This could mean that shame has different elicitors and effects on subsequent behaviours in these cultures, even in early development. Similarly, blushing involves the same physiological mechanism (increased blood flow and temperature of the cheek), but its visibility differs across individuals and cultures [108]. Thus, while the physiological process involved in blushing may be informative and functional for the blusher (e.g. in alerting them that they committed or are about to commit a social violation) no matter their culture, it is not yet clear whether blushing that is less visible to observers has the same social effects. Cross-cultural studies, including individuals with diverse skin tones, are therefore needed to understand whether blushing can be seen as a reliable indicator of embarrassment across cultures.

In addition, the reviewed studies provide inconclusive evidence regarding gender differences in the expression of self-conscious emotions. Some findings suggest that young girls show more shame, consistent with evidence that girls display more internalizing emotions than boys [109], whereas no consistent gender differences have been found for guilt, embarrassment and pride. This pattern is also in line with a meta-analysis of gender differences in self-conscious emotional experience in adults, which found only small gender differences in shame and guilt, but not in embarrassment and pride [110]. Gender stereotypes, however, maintain that women experience and display more shame, guilt and embarrassment [110], and in some childhood circumstances, such as living with and caring for depressed parents, girls may be more prone to experiencing guilt and shame and may become overinvolved at their own expense [111]. Future work should therefore systematically examine potential gender differences in self-conscious emotions, with particular attention to clarifying how socialization and other contextual factors shape the expressions of these emotions in boys and girls across early development.

Finally, although many studies used naturalistic tasks to evoke spontaneous emotional expressions, the majority have been conducted in laboratory settings. This may reduce ecological validity, raising the question of whether the same results would be found in more naturalistic settings, such as homes and day care centres. Naturalistic environments may present less controlled but more complex and rich interactions and behaviours, potentially revealing self-conscious emotions in a wider range of situations and earlier in development compared with laboratory settings. Therefore, observational research in more naturalistic settings is also required.

## 6. Conclusion

Research on self-conscious emotions in early development has blossomed in the past decades, but it remains relatively limited compared to research on other distinct emotions and cognitive development. Existing studies suggest that embarrassment, guilt, shame and pride emerge in the first years of life, and already play a role in motivating young children's behaviours in social situations. This underlies their importance for healthy social development. At the same time, the current evidence is still limited and often mixed, with many conclusions resting on a limited number of mostly correlational studies, using diverse methods and tasks. The development of new methods and techniques now makes it possible to study these emotions in non-verbal or just-verbal children by capturing, in a reliable way, non-verbal expressions and physiological responses accompanying these emotions. Therefore, future efforts by developmental and affective scientists should focus on experimental and longitudinal designs that can clarify causal mechanisms and developmental trajectories, systematic comparisons across elicitors, ages and components of emotions, and studies in diverse cultural contexts and naturalistic settings. Such efforts will be crucial for establishing when self-conscious emotions emerge in daily life, how they shape children's social relationships and how they can be leveraged to support healthy social development.

## Data Availability

This article has no additional data.

## Theme

One contribution of 19 to a theme issue ‘Mechanisms, development, phylogeny and functions of emotional expressions’.
